# Age-dependency in binocular rivalry is reflected by exclusive percepts, not mixed percepts

**DOI:** 10.1038/s41598-019-55890-5

**Published:** 2019-12-17

**Authors:** Elahe Arani, Raymond van Ee, Richard van Wezel

**Affiliations:** 1 Biophysics Department, Donders Institute for Brain, Cognition and Behaviour, Radboud University, 6525AJ Nijmegen, The Netherlands; 2 Department of Brain and Cognition, Leuven University, BE-3000 Leuven, Belgium; 3 Department of Brain, Behavior and Cognition, Philips Research, Eindhoven, The Netherlands; 4 Biomedical Signal and Systems Group, MIRA Institute for Biomedical Technology and Technical Medicine, University of Twente, Enschede, The Netherlands

**Keywords:** Cognitive ageing, Perception, Neural ageing

## Abstract

Some aspects of decision-making are known to decline with normal aging. One of the known perceptual decision-making processes which is vastly studied is binocular rivalry. It is well-established that the older the person, the slower the perceptual dynamics. However, the underlying neurobiological cause is unknown. So, to understand how age affects visual decision-making, we investigated age-related changes in perception during binocular rivalry. In binocular rivalry, the image presented to one eye competes for perceptual dominance with the image presented to the other eye. Perception during binocular rivalry consists of alternations between exclusive percepts. However, frequently, mixed percepts with combinations of the two monocular images occur. The mixed percepts reflect a transition from the percept of one eye to the other but frequently the transitions do not complete the full cycle and the previous exclusive percept becomes dominant again. The transitional idiosyncrasy of mixed percepts has not been studied systematically in different age groups. Previously, we have found evidence for adaptation and noise, and not inhibition, as underlying neural factors that are related to age-dependent perceptual decisions. Based on those conclusions, we predict that mixed percepts/inhibitory interactions should not change with aging. Therefore, in an old and a young age group, we studied binocular rivalry dynamics considering both exclusive and mixed percepts by using two paradigms: percept-choice and percept-switch. We found a decrease in perceptual alternation Probability for older adults, although the rate of mixed percepts did not differ significantly compared to younger adults. Interestingly, the mixed percepts play a very similar transitional idiosyncrasy in our different age groups. Further analyses suggest that differences in synaptic depression, gain modulation at the input level, and/or slower execution of motor commands are not the determining factors to explain these findings. We then argue that changes in perceptual decisions at an older age are the result of changes in neural adaptation and noise.

## Introduction

For successful visual perception, we need to interpret our surrounding visual world containing competing and contradictory information. Binocular rivalry is a visual decision process that is used already for a long time to study decision making mechanisms under these conditions^1,2^. Binocular rivalry occurs when two incompatible monocular stimuli are presented to the two eyes. Such dichoptic presentation typically causes oscillations in visual perception from one monocular image to the other^3–5^. In between these exclusive percepts, observers may report mixed percepts of a blend of the two eyes’ images^6–8^. Differences in occurrence of exclusive and mixed percepts under different stimulus conditions for different ages might reveal mechanisms of visual decision-making of the aging brain.

Models of binocular rivalry typically suggest that percept oscillations and mixed percepts are the result of interactions between the following neurobiological factors: neural adaptation, cross-inhibition between the two monocular neural populations, and noise in the neural system^9–12^. Each representation of rivalry stimuli is governed by two monocular neural populations coding for the stimuli presented in the two eyes. When activation of one population becomes stronger, those neurons determine the dominant percept. This stronger activation simultaneously inhibits the firing rate of the population of the competing representation^13^. The firing frequency of dominant neural activity is attenuated by spike frequency adaptation which can lead to dominance in the neural population for the other percept^14^. This process then continues with a stochastic signature. However, solely inhibition and adaptation cannot explain the stochasticity in the dynamics of binocular rivalry. This indicates that noise is a crucial force in rivalry^8,15^.

Mixed percepts consist of either superpositions of both eyes’ images or patchwork-like areas of local monocular dominance (“piecemeal rivalry”). In neural models, the difference between the activity of the rivaling monocular populations determines the level of exclusivity in visual perception^11^. Perception is exclusive if one population is active while the other population is silent, alternatively, when this difference is relatively small, perception becomes mixed. In line with experimental data, it has been shown that increasing the inhibition efficacy in neural models results in higher levels of exclusivity, whereas decreasing the inhibition efficacy causes lower levels of exclusivity^16^. It has also been reported that differences in mixed percepts are related to differences in mutual inhibitory interactions between two populations that represent the two percepts under binocular rivalry^16,17^. This has only been studied experimentally in autism^18^, in which autistic adults, in addition to slower alternation Probability, had a higher proportion of mixed percepts.

Binocular rivalry has been extensively used in studying a wide range of neural phenomena, including attention, perceptual memory, and consciousness in visual processing^19–22^. However, little attention has been paid to the effect of aging. A few studies showed that the rate of rivalry decreases in older adults (subjects 40–93 years^23^ and subjects 20–64 years^24^), and increases in children (in three age groups: 9-years, 12-years, and 21-years^25^). A generally accepted theory posits that the faster rate of rivalry is associated with a higher percentage of mixed percepts^26^, which is caused by a decrease in the inhibition efficacy, but this has not been tested experimentally. On the other hand, another theory of binocular rivalry postulates that the faster alternation Probabilities seen in children are linked to a larger and faster relative contribution of neural adaptation^25^. We have also recently hypothesised that changes in adaptation and noise, not inhibition cause age-dependency in perceptual decision^27^. Therefore, we predict that mixed percepts should not change with aging. Here, we study the effect of age on binocular rivalry, considering exclusive and mixed percept, to test our hypothesis and shed light on the neural mechanisms of visual perception.

Studying the role of each of the aforementioned neural factors (i.e., inhibition, adaptation and noise) in isolation is challenging. Here, we employ two different experimental conditions (continuous and intermittent presentations) to unravel the role of these factors. During continuous presentation, the rivalry stimulus is steadily presented and an observer perceives switches. While during intermittent presentation, the rivalry stimulus is presented for a shortened presentation duration interrupted by an inter-stimulus interval with a blank scene and an observer reports percept-choices during each stimulus presentation. It has been demonstrated that the underlying dynamics of percept-choices and percept-switches are different^11,28–31^. For percept-switches, switches occur when small noise signals trigger the opposite percept while the dominant percept diminishes its stability due to adaptation. Noest and colleagues showed that percept-choices depend crucially on an interaction between adaptation and a neural baseline activity. Adaptation and bias of both percept neural populations determine the percept-choices^11^. It has been shown that intermittent presentation at longer inter-stimulus intervals (>2 sec) evokes no alternations in rivalry perception^19,32^. The effect of adaptation fades away at longer inter-stimulus intervals and the dominant percept recovers from adaptation incurred in its previous dominance phase and hence gains strength again. Therefore, percept switch (continuous presentation) and percept choice (intermittent presentation) paradigms and different onset times/inter-stimulus intervals can control the dynamics of repetition, alternation, and more complex percept sequences, as well as reveal effects of cross-inhibition, adaptation, and noise.

**Figure 1:**
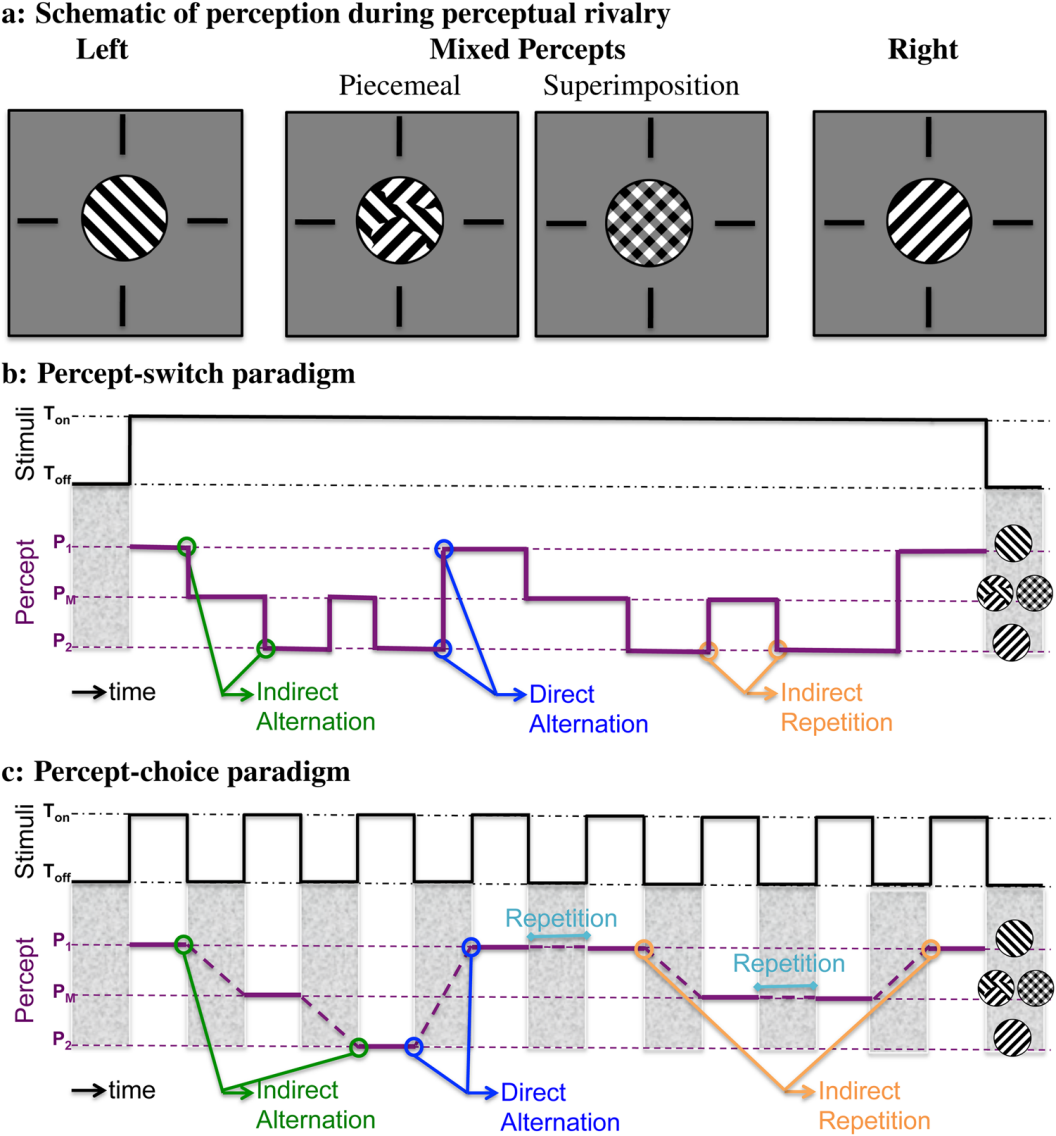
(**a**) Schematised binocular rivalry stimuli used in perceptual experiences during continuous and intermittent presentations. Each of the two presented images (one to the left and one to the right eye) can be perceived as an exclusive percept, or a mix of the two images, either spatially separated (piecemeal rivalry) or superimposed. (**b**) Experimental procedure for the percept-switch conditions where stimuli were presented continuously. The subjects reported the perceived percept (lower panel). A shift from one exclusive percept to the other exclusive percept is an alternation, the alternation can be perceived either directly (blue) or indirectly via mixed percepts (green). A shift from exclusive to mixed to the same exclusive percept is defined as an indirect repetition (orange). (**c**) Experimental procedure for percept-choice conditions where stimuli were presented intermittently (Ton) with an inter-stimulus period (Toff) where no stimulus was shown (upper panel). In addition to direct/indirect alternation and indirect repetition, two subsequent similar percept-choices can be perceived as repetition (cyan).

We here use a conventional orthogonal grating rivalry stimulus (Fig. 1a) in different experimental conditions (continuous and intermittent presentation of rivalry stimuli; Fig. 1b,c respectively). We demonstrate dominance (exclusive percept) duration, mixed percept duration, rate of indirect alternation, and rate of transition (indicator of an alternation rather than a repetition through a mixed percept). We also analyse chronic bias and reaction times, that could be related to eye deterioration and motor processing. For more detailed definition of these parameters please check the Methods section. We study these parameters in adults divided into a young (<31 years-old) and an old (>45 years-old) group. We decided to investigate short enough onset periods (1 sec) to prevent spontaneous percept switches during the stimulus presentation in intermittent presentations.

## Results

**Figure 2:**
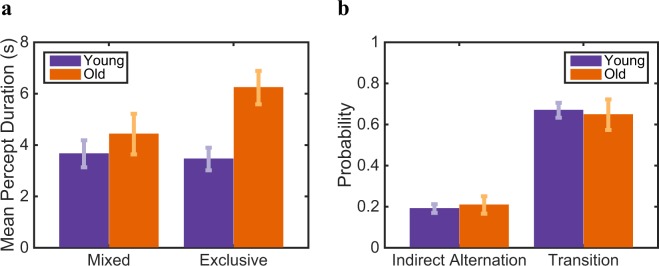
Age-dependency in percept-switch dynamics is prompted by exclusive percepts. (**a**) Mean percept duration of mixed and exclusive percepts, and (**b**) indirect alternation probability and transition probability during continuous presentation. The young group is in purple and the old group in orange. Error bars provide SEM. Only the mean percept duration of exclusive percepts differs significantly.

**Figure 3:**
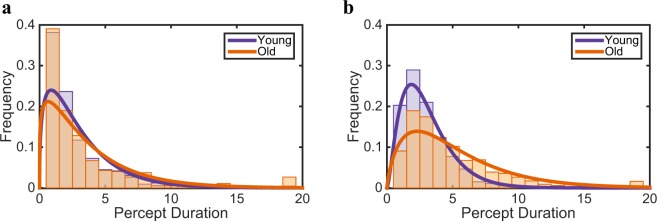
Shift in distribution of exclusive percept duration characterizes the age-dependency dynamics of percept-switches. Fitted Gamma distribution on duration of (**a**) mixed percepts, and (**b**) exclusive percept during continuous presentation. Young in purple and old in orange.

To investigate the role of inhibition, we analyze the *exclusive* and the *mixed* percept duration for two age groups in the percept-switch condition with sustained stimulation. Although, the mean percept duration of mixed percepts seemed to be slightly shorter for the younger group (Fig. 2a), this difference between the age groups is not significant (paired-samples t-test: *t*(21) = −0.82, *p* ≃ 0.42). There is also no significant difference in frequency of the occurrence of mixed percepts over time between our age groups (see SI, Fig. S2b, two-way ANOVA: *F*(17, 378) = 0.63, *p* = 0.87). In contrast, we found a significant difference between the young and old age groups in the mean of exclusive percept duration (paired-samples t-test: *t*(21) = −3.60, *p* < 0.001), where the percept duration gets longer for the higher age group. Further, we also calculated the probability of *indirect alternations*, which indicates the rate of alternation through mixed percepts, and the probability of *transitions*, which provides the tendency of mixed percepts to act as a transition state between exclusive percepts (Fig. 2b). The role of mixed percept does not change with higher ages, because the rate of indirect alternations and the rate of transitions does not change ((paired-samples t-test: *t*(21) = −3.38, *p* ≃ 0.70 and *t*(21) = 0.27, *p* ≃ 0.79, for probability of indirect alternation and probability of transition, respectively). Figure 3 depicts the histograms of the dominance intervals (defined as the periods of time during which one monocular stimulus is exclusively visible) and the mixed intervals, with a fit by Gamma distribution^33^. These results unanimously point out that age-dependent dynamics of percept-switches is mostly enforced by a difference in exclusive perception rather than mixed perception.

**Figure 4:**
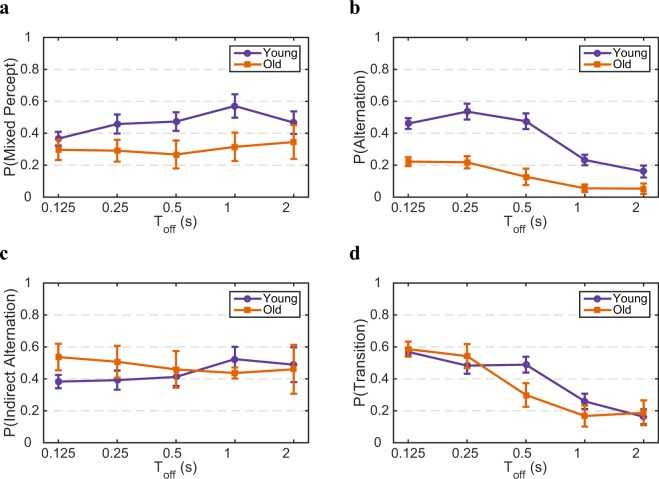
Exclusive percepts drive the age-dependency dynamics of percept-choices. (**a**) Mixed percept probability, (**b**) alternation probability, (**c**) indirect alternation probability, and (**d**) transition probability as a function of Toff during intermittent presentation. Young in purple and old in orange. Error bars provide SEM.

We then studied the role of adaptation and noise by investigating the effect of age on the dynamics of percept-choices using intermittent stimulation. We calculated the probability of mixed percepts, probability of alternations (ignoring the transitions), probability of indirect alternations (indicating the percentage of alternations that happens through mixed percepts), and finally the probability of transitions (indicating the preference of mixed percepts causing alternations and not repetitions). The data show that although there exists a slight decrease in the probability of mixed percepts for the old group, it does not decrease as Toff increases (*p* = 0.51), also age does not significantly affect this rate (Fig. 4a; *F*(4, 99) = 0.34, *p* = 0.85). Whereas, the perceptual alternation Probability declines for both age groups at longer Toff (*p* < 0.001), and age has a significant effect for this metric (Fig. 4b; *F*(4, 99) = 2.48, *p* < 0.05). Note that at the longer Toff’s the alternation probability curves of age groups converge, and the more distinguishable effect is at the shorter Toff’s. Notably, there is no decrease or increase in the probability of indirect alternation across Toff (*p* ≃ 0.99), and there is no effect of age on this probability (Fig. 4c; *F*(4, 85) = 0.58, *p* ≃ 0.68). Because longer Toff abolishes the effect of adaptation, this result disentangles the role of adaptation and inhibition, and conveys that the mixed percept/inhibitory does not affect the dynamics of alternations at different ages. Moreover, there is no age effect on the probability of transitions (the indication of a tendency that mixed percepts cause an alternation rather than a repetition), but this probability drops at longer Toff (Fig. 4d; *F*(4, 94) = 1.47, *p* ≃ 0.22). This result shows that the essence of mixed perception as a transition state between two exclusive percepts is changing by Toff for both age groups in the same manner. Therefore, the age-dependent dynamics of binocular rivalry is mostly affected by the exclusive perception, not mixed perception (cf. Fig. 4b,d).

**Figure 5:**
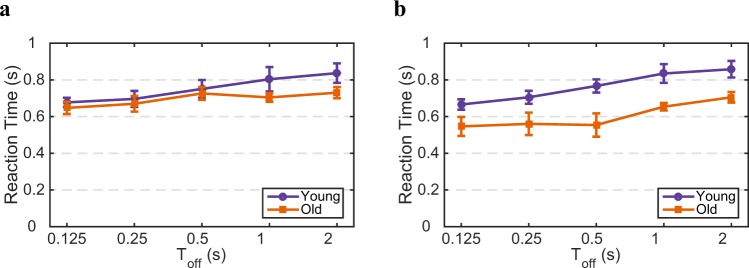
Age does not affect the overall reaction times during binocular rivalry. comparison between age groups of (**a**) mixed and (**b**) exclusive percepts shown in percept-choice conditions. The data do not reveal a significant difference in reaction times between the age groups for exclusive and mixed percepts during intermittent presentation across Toff. Young in purple and old in orange. Error bars provide SEM.

To control whether slower dynamics of perceptual decisions is affected by slower motor execution in older adults, we analyzed their reaction time from the onset of each trial in intermittent presentation. Older adults seem to have a faster reaction time, however, this effect is not significant for the interaction between both age and Toff for mixed (ANOVA: *F*(4, 88) = 0.53, *p* ≃ 0.71; Fig. 5a) and exclusive (ANOVA: *F*(4, 98) = 0.21, *p* ≃ 0.93; Fig. 5b) percepts. If one only considers the age, this effect for mixed percept is not significant (*p* ≃ 0.36) but older subjects have significantly faster reaction time for exclusive percepts (*p* < 0.01), and this is indeed an interesting and counterintuitive finding. However, we do not think this can explain the faster switch rate and alternation rate for younger subjects. Note that both age groups get significantly slower with longer Toff duration (*p* < 0.001). Similarly, reaction times in continuous presentations do not change significantly with age (paired sample t-test: *t*(67) = −0.8288, *p* ≃ 0.41).

**Figure 6:**
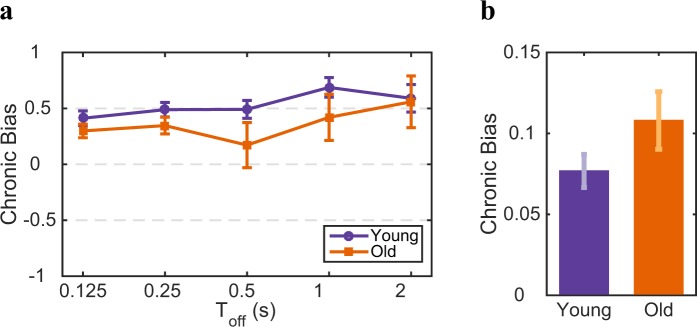
Age-dependency in the dynamics of binocular rivalry and chronic bias. Chronic bias refers to an intrinsic bias for a given perceptual state. Comparison between two age groups in (**a**) intermittent presentation and (**b**) continuous presentation conditions. Error bars provide one SEM. The data do not reveal an age-related difference in chronic bias between the two age groups (in both intermittent and continuous presentation).

It is known that eyes can deteriorate with age, which could give rise to biased input gain from the two eyes, if deterioration for one eye is different from the other eye. To check whether the difference in the alternation Probability are related to chronic biases we analyzed those biases for our two age groups. The chronic bias indicates the right-eye bias irrespective of perceptual history^34^ (see Methods). A two-way ANOVA revealed a significant main effect of Toff (*F*(4, 114) = 10.9, *p* < 0.001). However, the interaction between both age and Toff is not significant (*F*(64, 114) = 0.82, *p* ≃ 0.75) which indicates that there is no significant effect of age on the chronic bias at different Toff durations (Fig. 6a). Furthermore, a two-sample t-test did not show any significant effect on the average chronic bias for the groups during the switch percept conditions (*t*(21) = −0.11, *p* ≃ 0.92; Fig. 6b). We conclude that the slower dynamics of binocular rivalry in older adults is highly unlikely to be induced by eye deterioration or a difference in input gain.

## Discussion

We studied binocular rivalry by examining the role of exclusive and mixed percepts in two age groups using two experimental paradigms, i.e. percept-choice and percept-switch. Our findings indicate that the dynamics of binocular rivalry do robustly alter with age. Older adults demonstrate a longer percept duration during percept-switch condition and lower alternation Probability during the percept-choice condition, but there is no change in mixed percept duration or Probability (Figs. 2 and 4). Since, the mixed percepts can be understood in terms of weakened inhibition^16,35,36^, the finding that there is no significant difference in the dynamics of mixed percepts during binocular rivalry for higher ages, rules out a deciding role for neural inhibition. We also showed that these age-dependent effects are not caused by a slower motor command execution or by eye-deterioration in older adults (see Figs. 5 and 6). These results supplement our previous findings^27^, where we showed a significant age effect on coefficient of variation -the ratio of standard deviation to the mean of percept durations- which indicates the role of neural adaptation and noise. The difference that we report in this study could maybe have been larger if we would have compared a much younger group with a much older group of subjects. Our current and previous results lend support to our hypothesis that it is not the change in inhibition, but shifts in neural adaptation and noise, that alter rivalry dynamics in the aged brain.

In most previous studies the occurrence of mixed percepts in binocular rivalry is neglected. However, mixed percepts could reveal characteristics of binocular rivalry mechanisms, such as the effect of age in this paper. If we re-analyze our data under the assumption that subjects can only report exclusive percepts (remove mixed percepts), we would have missed the significant difference in aging (see Fig. S3). This might also explain why we in our previous paper^27^, where subjects could only choose between exclusive percepts, did not find a significant effect of age on the mean percept durations for continuous presentation. We argue that measuring the’third’ (mixed) percept in binocular rivalry experiments is important when comparing different groups of subjects.

It has been reported that an excitation-inhibition imbalance increases the proportion of mixed percepts and results into lower rivalry rates^16–18^. It has also been reported that weaker inhibition leads to shorter percept durations, hence slower growth of cumulative frequency of percept duration^37^. In rivalry models, exclusive dominance is considered as a product of strong and competitive inhibition between monocular views^11,12,16^ predicted that a reduction in the strength of such inhibition leads to an increase in mixed percepts. In this study, we show that there is no significant difference in the mean duration of mixed percepts during continuous presentation (see Fig. 2a), the probability of mixed percepts during intermittent presentation (see Fig. 4a) and the cumulative distributions of percept durations (see Fig. S2a) with age. Yet, the rate of rivalry differs significantly for different age groups. By calculating the *indirect alternation* probability and *transition* probability (Figs. 2b and 4c,d), we show that the duration of mixed percepts for the young and old groups is similar, which indicates that neural mechanisms that are specifically involved in mixed percepts (inhibitory interactions) may not be responsible for the difference in percept durations that we report between age groups. These analyses imply that the level of inhibition in our age groups does not differ meaning that inhibition can not be considered as the cause of age-dependency in binocular rivalry. Moreover, we show that the frequency of mixed percept in a prolonged presentation does not change for any of the age groups with the passage of time (Fig. S2b). However, the exclusive percept duration and alternation probability change for different age groups. Altogether, these results imply that the neural mechanism of mixed percepts which is known as a transitional state between two exclusive percepts^8,38^ is not the driving force behind age dependent perceptual decisions.

Our results show that the aging effect can not be trivially explained by changes in reaction time or chronic biases (see Figs. 5 and 6). In our measurements we did not control for eye movements and/or attention, so we cannot exclude that there is a difference for the two age group for those factors, but from the literature^39,40^ and our measurements we have no indication that eye movements or attentional strategies to perform the task are different for the two groups.

Many models have been proposed in the area of binocular rivalry or bistable stimuli, e.g.^9,11,12,41,42^. Most of these models explain rivalry as read-out from two pools of competing (cross-inhibiting) and adapting neurons that are also influenced by noise. It is not straightforward to study the role of these three factors separately, because they can interact dynamically. Inhibition^43^, adaptation^25^, and internal noise^44–46^ are known to change with age. For instance, human psychophysical experiments have shown that noisy adaptation dynamics generates a gamma-distribution of intervals^47^. Due to the strong correlation between age and the factors that determine binocular rivalry^8^, further studies on age-dependency in dynamics of binocular rivalry, could provide more information regarding the aging brain and a better understanding of the underlying mechanisms of perceptual decisions.

## Methods

### Visual stimuli

The size of the rivalry stimuli was about 2.4 degrees in diameter. The stimuli were presented on a 22 inch CRT screen (LaCie Electron 22 blue IV) with a resolution of 1600 × 1200 pixels (size of 390 × 295 mm) and a refresh rate of 100 Hz. Black and white gratings were masked with a Gaussian filter (*σ* = 0.5 degree) to fade out the borders. The rivalry stimuli (Fig. 1a) had a spatial frequency of 1.75 cycles/degree and were tilted clockwise (right eye) and anti-clockwise (left eye) with an angle of plus or minus 45 degrees. These orientations were constant during the whole experiment. Subjects viewed the stimulus through a mirror stereoscope in a dark room, at a distance of 100 cm from the screen. Subjects reported the orientation of the perceived rivalry gratings by using the left or right arrow on a keyboard. Subjects were instructed to choose between three different percepts by pressing either one of the keys for the corresponding orientation, and press both keys simultaneously to report mixed percepts. The rivalry stimuli were surrounded at a distance of 1.45 degrees by cross-hairs (0.975 × 0.062 degrees) to help and improve proper binocular fusion. The experiments controlled by custom-made software in MATLAB (The MathWorks Inc. 2014b) using the PsychToolbox_3.

### Subjects

Twenty-three subjects (13 female, 12 young) participated and were split into two age groups (young = 21.5 ± 3.0 and old = 59.5 ± 6.1 years-old). Subjects were recruited from university students, employees, and retired employees and via advertisements in local newspapers. Subjects had normal (or corrected to normal) vision. Subjects were instructed to adjust the orientation of the dichoptic mirrors of the stereoscope until the two images were aligned entirely in a comfortable way.

All subjects gave their full written informed consent prior to their participation. The local Ethics Committee of the Faculty of Social Sciences of the Radboud University (ECSW) approved the experimental protocols (ECSW2016-2208-41). All experiments adhered to the relevant guidelines and regulations for which ethical approval was obtained.

### Experimental procedure

The experiment was set up in different blocks of two conditions: continuous presentation (percept-switch) or intermittent presentation (percept-choice). Instructions were presented on the screen between the blocks to inform the subjects about the upcoming block condition. In continuous presentation blocks, subjects were asked to respond each time the observed image switched from one to the other percept. There were three blocks of 3 minutes duration. In intermittent presentation blocks, subjects responded each stimulus presentation. Five different inter-stimulus durations (Toff) were used (125, 250, 500, 1000 and 2000 ms) with a fixed 1 sec stimulus duration (Ton). Each condition was repeated two times and the duration of each block was 2 minutes.

Together, this resulted in six different conditions and a total duration of the whole experiment of 29 minutes. Before the experiment, subjects performed a two-minute test session in order to check whether they could perceive and report the perceived orientations correctly.

### Data analysis

No discrimination was made between different types of mixed percept (piecemeal or superimposed rivalry). Left and right key presses with less than 150 ms intervals were interpreted as a mixed response. Two subsequent different exclusive percepts is defined as an alternation, either *direct alternation* or *indirect alternation* via one or more mixed percepts.

In continuous presentation blocks, the average percept duration was calculated, which is referred to as the *mean percept duration* (Fig. 1b). Two consecutive identical key presses were interpreted as one key press. The last key-response of each block was ignored. In intermittent presentation blocks, subjects were instructed to respond only once during the one-second stimulus presentation duration (Fig. 1c). In case of multiple key-responses within the 1 second stimulus, the first response was selected and the second ignored (on average less than 2% of multiple response per subject took place, and more than 57% of multiple responses were the same). Trials without a response by the subject were excluded, together with the preceding an subsequent trial.

The definition of the used probabilities:

- **Mixed percept probability**- The ratio of number of reported mixed percepts to the total number of responses:
  1 $$p(Mixed\,Percept)=\frac{\#mixed\,responses}{\#total\,responses}$$
- **Alternation probability**- The ratio of interchanges between two exclusive percepts (i.e., sum of direct and indirect alternations) to all reported exclusive percepts (i.e., sum of alternations and repetitions):
  2 $$p(Alternation)=\frac{\#alternations}{\#exclusive\,responses}$$
- **Indirect alternation probability**- The ratio of alternations through mixed percepts to total number of alternations (total number of switches between two exclusive percepts by ignoring the mixed percepts):
  3 $$p(Indirect\,Alternation)=\frac{\#indirect\,alternations}{\#alternations}$$
- **Transition probability**- The probability that indicates how much a mixed percept causes an alternation rather than a repetition:

4 $$p(Transition)=\frac{\#indirect\,alternations}{\#indirect\,alternations+\#indirect\,repetitions}$$

#### Reaction time

In percept-switch conditions, we could also analyze the reaction time of subjects. The duration from the onset of each trial to reported response defined as reaction time.

#### Perceptual index

In this experiment, we also analyzed data based on a perceptual index (called chronic bias), which have been described previously^34^. This index ranges from −1 to +1 and is zero when there is no effect of its measurement. The property of single percepts is defined as the chronic bias (denoted by c):

5 $$c=p({P}_{2})-p({P}_{1})$$

#### Statistics

To test for group differences in intermittent presentation, a mixed ANOVA with factors of group (young vs. older), and inter-stimulus duration (Toff) as within-group factor were conducted on mixed percept, (indirect) alternation, and transition probability values as well as on reaction and chronic bias. Also, the analysed mixed percept duration, exclusive percept duration, indirect alternation duration, transition duration, chronic bias, and reaction time during continuous presentation for all subjects were eventually compared between the two groups using paired t-test. The Statistics and p-values are reported accordingly for each test.

To test the regression coefficients of CV (coefficient of variations, generalized linear F-test is applied^48^. Using an F-statistic, it shows whether or not to reject the full model (pooled all subjects) in favor of the reduced models (separate models for each group). We reported the according p-value for mixed and exclusive percepts.

To test the statistical difference of cumulative frequencies of percept duration between our age groups, we performed two-sample Kolmogorov-Smirnov test. It tests the null hypothesis that data in old and young percept durations comes from populations with the same distribution, against the alternative hypothesis that the cumulative frequency (cdf) of the distribution of old percept durations is larger than the umulative frequency of the distribution of young percept durarion.

## Supplementary information

Supplemental Information

## Data Availability

The experimental data can be obtained from the corresponding author upon reasonable request.
