# Supplementary material for: Age-dependency in binocular rivalry is reflected by exclusive percepts, not mixed percepts
Source: Sci Rep. 2019 Dec 17;9:19271. doi: 10.1038/s41598-019-55890-5 (PMC6917811; doi:10.1038/s41598-019-55890-5)
Supplement: Supplementary file 1 — Supplemental Information [file 41598_2019_55890_MOESM1_ESM.pdf]

Age-dependency in binocular rivalry is reflected by exclusive percepts, not mixed percepts

Elahe Arani, Raymond van Ee, Richard van Wezel

Supplemental Information

Here, we analyze the effect of noise for both mixed and exclusive percepts during percept switch condition. Noise effect is measured using coefficient of variation which is the ratio between standard deviation and mean of percept durations. This ratio ranges from 0 (pure adaptation, no noise) to 1 (pure noise, no adaptation). We used a statistical approach (generalized linear F-test, see Method) to find out whether age has a significant role here. Coefficient of variation is significantly different only for the mixed percept ($p < 0.05$; Figure S1a), not for exclusive percept ($p = 0.55$; Figure S1b).

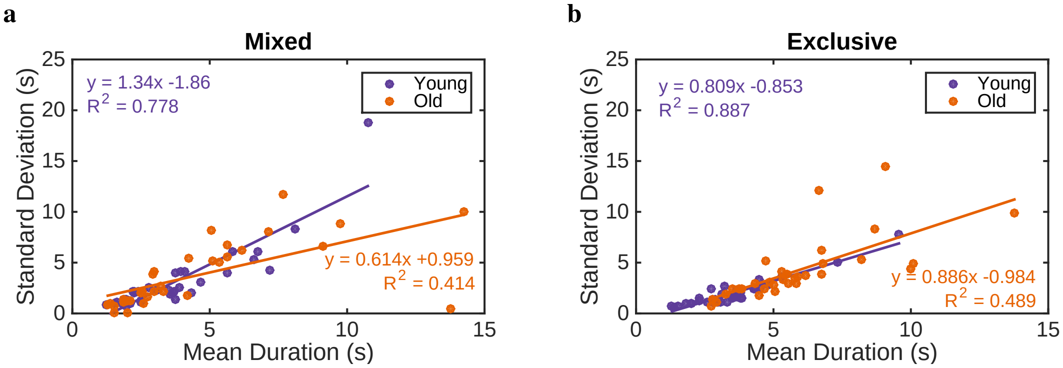

Figure S1. Effect of noise: The coefficient of variations of percept durations during percept-switch for (**a**) mixed and (**b**) exclusive percepts.

It is know that more inhibition causes longer percept durarions[1,2]. Then, we showed the cumulative frequency of percept duration during continuous presentation for both age groups (Figure S2a). There is no significance difference between the cumulative frequency of old and young age group ($p \simeq 0.12$, $ks2stat = 0.1833$). This indicates that Kolmogorov-Smirnov test rejects the null hypothesis, in favor of the alternative hypothesis that the cumulative frequency of percept durion in old group is larger than the cumulative frequency of percept durion in young group, at the default 5% significance level.

We examined the effect of age on mixed percepts over time (Figure S2b). For that purpose, we divided the continuous blocks to the discrete bins of 10 seconds duration and counted the number of reported mixed percepts for each subject. Once more, we did not find a significant difference between the number of mixed percepts in our age group over time ($p \simeq 0.57$), however, these numbers per bin are slightly lower in the older age group for all bins. Note that the number of mixed percepts does not change noticeably over time. In conclusion, the underlying neural mechanism of mixed percepts can not cause the age-dependent dynamics of binocular rivalry.

In many studies, subjects are given only to choose between two choices (called two-alternative forced choice experiment), which force them to report the most dominant percept. To check how the brain convert a three-choice to a two-choice condition, we converted the reported mixed percept to exclusive percept following this rule: (i) if the mixed percept occurs in between two different exclusive percepts, randomly converted to left or right, (ii) otherwise, if the mixed percept occurs in between two similar percepts, it converted to the same one. In both percept-switch and percept-choice conditions, the mean percept duration and the alternation Probability have undergone changes, because the dynamics are slowed down due to converting mixed percept to one of the dominant percepts (Figure S3). Although, the increase of mean percept duration is slightly more for older adults (%82 vs %71 for younger adults), still the durations are in the experimentally observed range[3]. However, in this analysis, age did not have a significant effect on the mean percept duration for similar age groups which is in line with the recent findings[3]. Interestingly, this transformation does not change the shape of alternation Probability curve in percept-choice condition and the result of analysis is very similar to the mentioned manuscript.

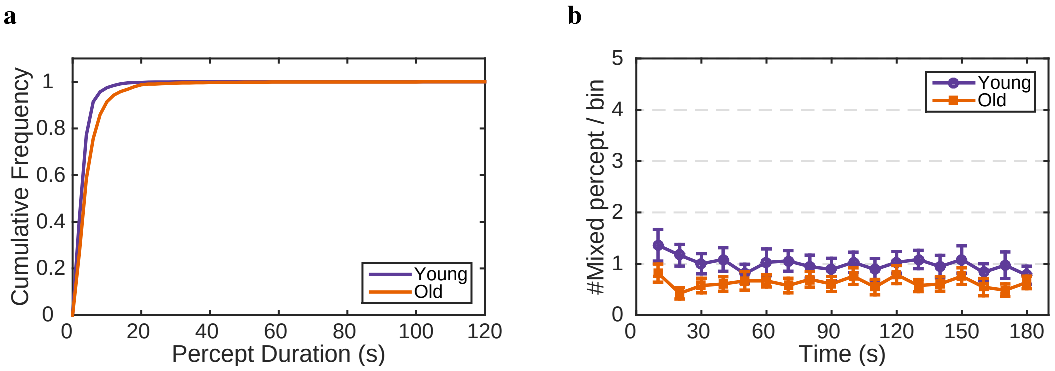

Figure S2. Percept-switch: (**a**) the cumulative frequency of percept durations and (**b**) the average number of mixed percept per bin of 10 seconds duration in the continuous presentation for the young (purple) and old (orange) groups. Error bars provide SEM.

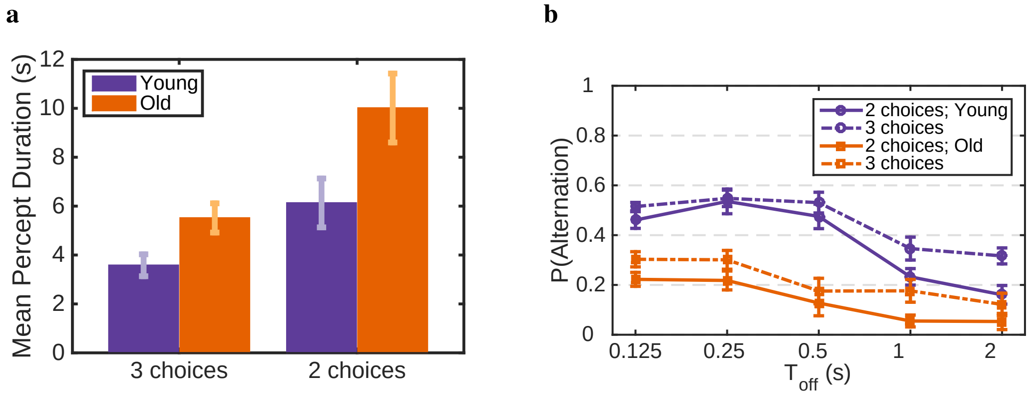

Figure S3. Three-choices vs two-choices: mean percept duration of all three choices and converting to two-forced-choices in (**a**) continuous and (**b**) intermittent presentations. Young in purple and old in orange. Error bars provide SEM.

References

1. Hoshino, O. Ambient gaba responsible for age-related changes in multistable perception. *Neural computation* **25**, 1164–1190 (2013).
2. van Loon, A. M. *et al.* Gaba shapes the dynamics of bistable perception. *Curr. Biol.* **23**, 823–827 (2013).
3. Arani, E., van Ee, R. & van Wezel, R. Changes in low-level neural properties underlie age-dependent visual decision making. *Sci. reports* **8**, 10789 (2018).
